# Supplementary material for: The Ocean’s Pharmacy: Health Discoveries in Marine Algae
Source: Molecules. 2024 Apr 22;29(8):1900. doi: 10.3390/molecules29081900 (PMC11055030; doi:10.3390/molecules29081900)
Supplement: Supplementary file 1 [file molecules-29-01900-s001.zip › molecules-2924792-supplementary.pdf]

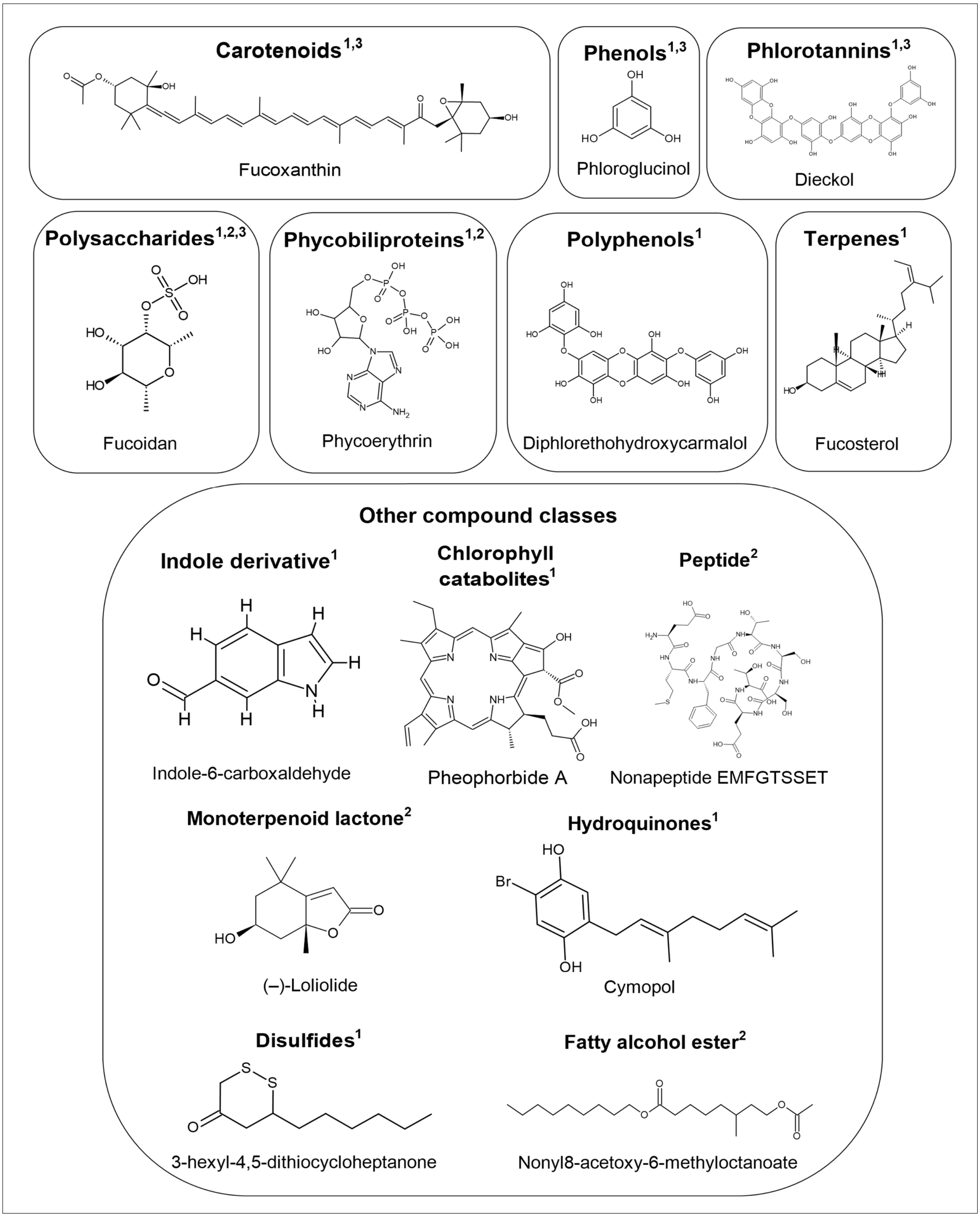

Figure S1. Chemical classification of isolated algal compounds used in cell and *in vivo* studies. Isolated compounds studied were classifiable in 7 categories: polysaccharides (n=37), phlorotannins (n=15), terpenes (n=12), carotenoids (n=6), polyphenols (n=4), phenols (n=3) and other compound classes (indole derivates, chlorophyll catabolites, peptide, monoterpenoid lactone, hydroquinoes, disulfides and fatty alcohol ester with n=1 each). The depicted compounds represent the most prevalent in each chemical class, based on the findings from the included cell and in vivo studies in this review. Superscript numbers (1,2 and 3) refer to the source of the isolated compounds: (1) macroalgal, (2) microalgal or (3) not-defined algae origin, respectively.
